# Atypical musculoskeletal manifestations on flexor hallucis longus tendon of gout causing tarsal tunnel syndrome in diabetic patients: A case report

**DOI:** 10.1097/MD.0000000000018374

**Published:** 2019-12-20

**Authors:** Yoon Seok Kim, Min Ki Lee, Young Yi

**Affiliations:** Department of Orthopedic Surgery, Seoul Paik Hospital, Inje University, Seoul, Republic of Korea.

**Keywords:** case report, flexor hallucis longus tendonitis, gout, tarsal tunnel syndrome

## Abstract

**Rationale:**

Deposition of tophus is a common feature in chronic gout; however, signs and symptoms are not always well-pronounced in cases of uncommon sites. We report a rare case with a tophaceous tendonitis on the flexor hallucis longus (FHL) tendon with tarsal tunnel syndrome (TTS). This is the first surgical case of TTS by gouty tophi in FHL.

**Patient Concerns:**

A 55-year-old woman presented with a 6-month history of mild discomfort at the right foot, which gradually worsened in the past 3 weeks. The patient visited our outpatient clinic due to persistent and aggravating foot pain and swelling around the tarsal tunnel.

**Diagnosis:**

The patient was diagnosed with hyperuricemia and diabetes mellitus with chronic kidney disease, and did not receive regular antigout treatments. Paresthesia was found along the distribution of medial and plantar nerve and tinel test was positive on tarsal tunnel. Biochemical examination showed she had raised serum uric acid (10.6 mg/dL) and decreased estimated glomerular filtration rate (69 mL/min/1.73 m^2^). Conventional radiography examination showed negative pathology except soft tissue swelling. Magnetic resonance imaging revealed a fusiform mass within the FHL tendon and fluid collection around tarsal tunnel.

**Interventions:**

Surgical exploration was performed to remove the mass. Inflammation fluid exploded out from FHL tendon sheath, which was later proven to have infiltration of monosodium urate crystal. Superficial dissection revealed a white chalky mass and posterior tibial nerve was significantly compressed by the tophus mass.

**Outcomes:**

The mass was removed and the symptoms were relieved at immediate postoperative period.

**Lessons:**

A tophaceous tendonitis on FHL tendon can cause TTS and surgical decompression of the gout lesion can reduce the symptoms.

## 1 Introduction

Many studies report that the incidence of gout increases continuously for several decades.^[1]^ Although the gouty tophus is more commonly involved in Achilles and patellar tendon, some gouty tophi are found in the carpal tunnel and tendons of the upper limb.^[2–5]^ There has, however, been no case of tophaceous gouty tendonitis found in the flexor hallucis longus (FHL) tendon in the distal part of the lower limb. Also, there are only few case reports of tarsal tunnel syndrome (TTS) due to gout tendonitis.

TTS is an entrapment neuropathy of the posterior tibial nerve or one of its branches within the tarsal tunnel. Many etiologies have been described and broadly classified as trauma, space-occupying lesions, foot deformities, etc.^[6]^

The tarsal tunnel is an area of anatomic narrowing caused by tight ligamentous structures. It is a fibro-osseous tunnel bordered superficially by the flexor retinaculum, which passes obliquely from proximal to distal to anterior.^[7]^ In a literature review of TTS, it was estimated that in 60% to 80% of cases, the specific etiology can be identified.^[6]^

We report a case of TTS caused by a tophaceous gout tendonitis with monosodium urate (MSU) crystal deposition at the FHL tendon that has been resolved with resection of tophus mass and tendon sheath.

## 2 Case presentation

Patient has provided informed consent for publication of the case. A 55-year-old woman presented in our outpatient clinic with mild discomfort at the right foot since 6 months ago, which gradually worsened in the past 3 weeks. Her discomfort was mainly pain with ankle movement and numbness which had been not relieved by nonoperative treatments, including activity modification, physical therapy, and oral anti-inflammatory medicine. There was no previous history of trauma, but she had swelling around the tarsal tunnel.

Patient had a medical history of asymptomatic hyperuricemia (highest serum uric acid level was 10.6 mg/dL, untreated) and diabetes mellitus (routinely treated with oral antihyperglycemic drugs). Patient was also noted to have decreased eGFR (69 mL/min/1.73 m^2^; mildly decreased renal function according to the Chronic Kidney Disease Epidemiology Collaboration calculation).

On physical examination, paresthesia was found along the distribution of medial and plantar nerve. She walked with an antalgic gait for plantar heel pain especially when she wore a hard soled shoe on the right foot. Tinel sign was positive and tenderness was positive along with FHL tendon pathway.

The patient was then sent for nerve conduction studies and the study showed posterior tibial nerve entrapment at the tarsal tunnel area. But there was no abnormality found in the electromyography study.

**Figure 1:**
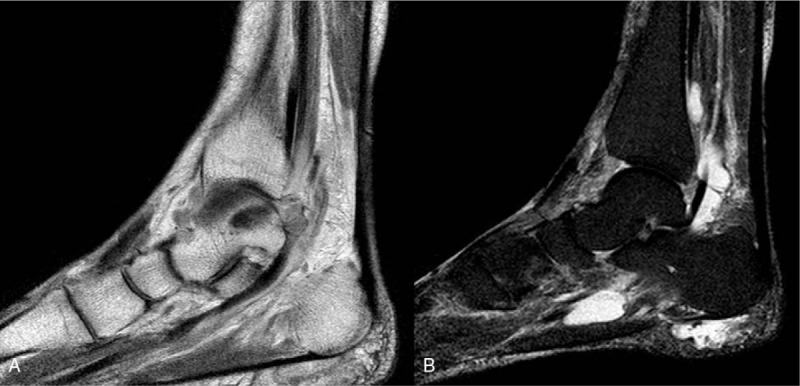
Sagittal T1-magnetic resonance imaging (MRI) image of tophus mass on the tarsal tunnel area. A, Sagittal T2 MRI image of tendonitis with fluid collection around flexor hallucis longus (FHL) tendon, adductor hallucis longus, and insertion of the plantar fascia.

Conventional radiography examination of the ankle showed negative pathology except soft tissue swelling. Magnetic resonance imaging revealed a fusiform mass, which was heterogeneous isointense on sagittal T1-image within the FHL tendon. Fluid collection was detected around FHL tendon, adductor hallucis longus tendon, and calcaneal attachment of plantar fascia and it was heterogenous hyperintense on T2 image (Fig. 1).

A diagnosis of TTS caused by space occupying lesion with fluid collection was made, taking gouty arthritis with tophi as the most suspicious cause of the problem. As pain with the neurologic symptom aggravated over 6 months and obvious space occupying lesion in tarsal tunnel was found, the patient decided to take a surgery for excision of the mass.

**Figure 2:**
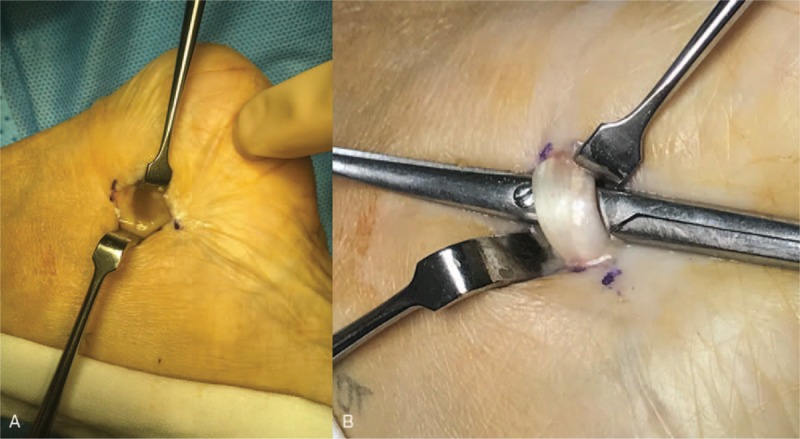
A, Inflammation fluid exploded out from flexor hallucis longus (FHL) tendon sheath. B, Infiltration of a white chalky mass on the FHL tendon.

**Figure 3:**
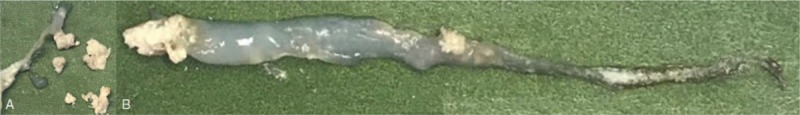
A, Excised gout tophi and flexor hallucis longus (FHL) tendon sheath. B, FHL tendon sheath containing gout tophi.

Surgical exploration was performed to remove the mass using a minimized conventional medial approach, making incision along the tarsal tunnel extending proximally. Inflammation fluid exploded out from FHL tendon sheath which infiltrated in MSU crystal (Fig. 2A). Superficial dissection revealed a white chalky mass, MSU crystal had infiltrated the FHL tendon (Fig. 2B). Posterior tibial nerve was significantly compressed by the gouty tophus. The mass did not adhere to the surrounding structures. The tophus mass was removed, and the FHL tendon sheath along with MSU crystal was excised (Fig. 3).

After the surgery, the plantar foot paraesthesia subsided immediately and the patient satisfied with the symptom relief at the early and medium term follow-up period. At 1 year after the surgery, she is still pain free and returned to her daily activities only with some restrictions for sport activities.

## 3 Discussion

This is the first case of TTS provoked by gouty tendonitis on FHL tendon. The symptoms improved obviously after surgical removal of the tophi and surrounding inflammatory tissues. Our case is rare in 2 aspects; the first aspect is that no article makes mention of gouty tendinosis on FHL tendon; the second one is that only few case reports refer to gouty tophus as a reason of TTS.^[8–10]^ Lui^[8]^ reported a case of TTS caused by gouty tophus. Although there were many similar features to our case, they specified that FHL tendon was not involved in their case and the symptoms were not satisfactorily relieved.

As we briefly mentioned at the introduction, TTS can be attributed to various etiologies that courses within the anatomic boundaries of the tarsal tunnel; varicosities, venous congestion, ganglia, perineural fibrosis, lipoma, neurilemoma, hypertrophic flexor retinaculum, hypertrophic or accessory abductor hallucis muscle, flexor digitorum accessory longus muscle, partial rupture of the FHL tendon, fluid retention, and chronic phlebitis.^[11–19]^ There are some etiologies that compromises the tarsal tunnel from outside; osseous prominences, postsurgical scarring, generalized lower extremity edema, hind foot deformities, and inflammatory disorders including rheumatoid arthritis, ankylosing spondylitis, and synovial osteochondromatosis.^[11–19]^ Each of the etiologies has been reported more commonly than the gouty tophus has been done, so that it is hard to make a suspicion on a gouty tendonitis as a culprit of TTS.

In our case, no significant past gouty attack, family history, or past medical history of hypertension and diuretics consumption was found except asymptomatic hyperuricemia and diabetes with chronic renal disease. These atypical initial findings could mislead the clinician not to arrive at the initial diagnosis of tophaceous gout.

There is lack of studies on the natural course of the TTS caused by tophaceous gouty tendonitis on FHL tendon, which could make it hard to decide a treatment strategy. Some authors have reported that early surgical treatment is recommended when the cause of TTS is identified such as a space occupying lesion, based on some studies suggesting poor outcome with delayed surgery.^[20,21]^ Predictable postoperative complications are poor wound healing, wound infection, recurrence of TTS due to tophaceous discharge, and postsurgical scarring. Surgeons are mandatory to take these complications into consideration while planning the treatment strategy.

## 4 Conclusion

We present an unreported cause of a space-occupying lesion in the etiology of TTS, namely the combination of a tophaceous gout. Atypical symptoms and findings of gouty tendonitis could lead doctors to misdiagnose the disease as TTS caused by a simple FHL tenosynovitis, and not to care about the gout. Through the case, we suggest that gouty tendonitis with tophi should be considered in diagnosing and treating TTS. After the diagnosis, surgical excision of tophi and surrounding inflammatory tissues can resolve the symptoms and following treatment for gout is also needed.

## Author contributions

**Conceptualization:** Min Ki Lee, Young Yi.

**Data curation:** Young Yi.

**Formal analysis:** Young Yi.

**Funding acquisition:** Young Yi.

**Investigation:** Young Yi.

**Methodology:** Young Yi.

**Project administration:** Young Yi.

**Resources:** Young Yi.

**Software:** Young Yi.

**Supervision:** Young Yi.

**Validation:** Min Ki Lee.

**Writing – original draft:** Yoon Seok Kim.
